# Lactylation and acetylation: parallel paths, divergent deeds, and research dilemmas

**DOI:** 10.1186/s12967-026-07877-w

**Published:** 2026-03-23

**Authors:** Wei Jiang, Yuanfei Chen, Shiwan Lin, Yanfang Liu, Xiang Liao

**Affiliations:** 1 Department of Laboratory Medicine, Affiliated Hospital of Jiangsu University, 438 Jiefang Road, Zhenjiang, Jiangsu Province 21001 China; 2 Laboratory Center, Affiliated People’s Hospital of Jiangsu University, No. 8, Dianli Road, Zhenjiang, Jiangsu Province 212002 China; 3 Institute of Medical Imaging and Artificial Intelligence, Jiangsu University, 438 Jiefang Road, Zhenjiang, Jiangsu Province 212001 China

**Keywords:** Lactylation, Acetylation, Metabolism, Acylation, Post-translational modifications

## Abstract

**Background:**

Metabolism-induced post-translational modifications (PTMs) are critical for the regulation of cellular activities. As important types of modifications, lactylation and acetylation play essential roles in health and disease. With the development of lactylation research, its regulatory mechanism has been revealed to be highly similar to that of acetylation.

**Main body:**

Most lactylated proteins are also acetylated. They share lysine modification sites and exert similar regulatory functions in gene transcription, DNA repair, signal transduction, autophagy, and metabolism, although certain differences exist. Both lactylation and acetylation regulate protein functions by affecting protein stability, enzyme activity, liquid-liquid phase separation, and crosstalk with other modifications. More importantly, the high similarity between their regulatory mechanisms brings challenges to their specific research and raises previously overlooked questions for published acetylation studies.

**Conclusion:**

This review discusses the regulatory mechanisms, functional differences, and research dilemmas of lactylation and acetylation.

## Introduction

Post-translational modifications (PTMs) of proteins are essential for the precise regulation of protein functions. Compared with the regulation of gene expression, metabolism-induced PTMs can regulate protein functions more promptly and accurately [1]. Currently, hundreds of types of PTM have been discovered, such as lactylation, crotonylation, isonicotinylation, 2-hydroxyisobutyrylation, etc., and new types of PTM are constantly being reported. Different from other modifications, lactylation and acetylation have gained attention due to sensitive regulation and the high density of substrates, lactate and acetyl coenzyme A (acetyl-CoA) [2].

Due to the Warburg effect, namely aerobic glycolysis, there is a high lactate concentration and acidic microenvironment inside the tumor [3]. In 2019, Zhang et al. first reported lactylation and discovered the anti-inflammatory effect of histone lactylation in macrophages [2]. Lactylation is the conversion of lactate (CH₃CH(OH)CO-) into lactyl CoA under the action of acyl transferases, which writes the lactate group to the corresponding sites (lysine) of proteins. The discovery of lactylation explains the physiological functions of lactate from a new perspective, such as immunosuppression, promotion of glycolysis, angiogenesis, DNA repair and cell cycle regulation, etc. The lactylation has three isomers: L-lactate-lysine (Kl-la), N-ε-(carboxyethyl)-lysine (Kce), and D-lactate-lysine (Kd-la) [4]. Kl-la is the primary lactylation isomer on histones and the main responder to glycolysis and the Warburg effect, while Kd-la or Kce are not. Besides L-lactate, the role of D-lactate in the human body has also been revealed [5]. In the following text, unless otherwise specified, lactylation refers to L-lactylation. With the development and application of lactylation peptide purification technology, lactylation proteomics (lactylome) has been extensively explored. As a result, thousands of histone and non-histone proteins undergoing lactylation have been identified across different models [6–8].

Acetylation is one of the classic and widespread PTM in cells. In 1964, Vincent Allfrey and his colleagues first discovered acetylation on histones, and defined acetylation as a chemical reaction that introduces an acetyl group (CH3CO-) into the molecules of organic compounds [9]. Protein acetylation can affect its conformation and charge distribution, thereby regulating gene transcription, the interaction of proteins with other molecules and the activity of enzymes [10]. As one of the classical PTMs, acetylation regulates signal pathways involve almost all functional phenotypes. Besides lysine-acetylation, acetylation encompasses N-terminal protein acetylation and O-linked acetylation of serine and threonine [11, 12]. Since lactylation has only been discovered at lysine sites so far, acetylation in this review refers specifically to lysine acetylation (Kac). Its reaction substrate, acetyl-CoA, is the metabolic hub in cells [13].

Both lactylation and acetylation fall under the category of acylation modifications. They occur at lysine residues and share common regulatory proteins, namely “Writers”, “Erasers”, and “Readers”. And some specific regulatory proteins stand out, such as the specific regulators of lactylation, Alanyl-tRNA Synthetase 1 and Alanyl-tRNA Synthetase 2 (AARS1 and AARS2) [14, 15]. Besides difference in regualtion, there are functional differences between lactylation and acetylation. For example, the choice of acetylation and lactylation in macrophages leads to the distinction between pro-inflammatory and anti-inflammatory phenotypes, but the specific mechanism of the regulation of this difference by cells remains unknown [2]. In the early studies of acetylation, due to the lack of identification of lactylation and other acylation modifications, methods such as modulating the acylation regulatory enzymes or mutating key sites were widely used. Until today, such similar experimental methods and results are still being used and published. This is hazardous because such data cannot directly reflect the function of some certain modification. A comprehensive review of lactylation and acetylation is crucial.

**Fig. 1:**
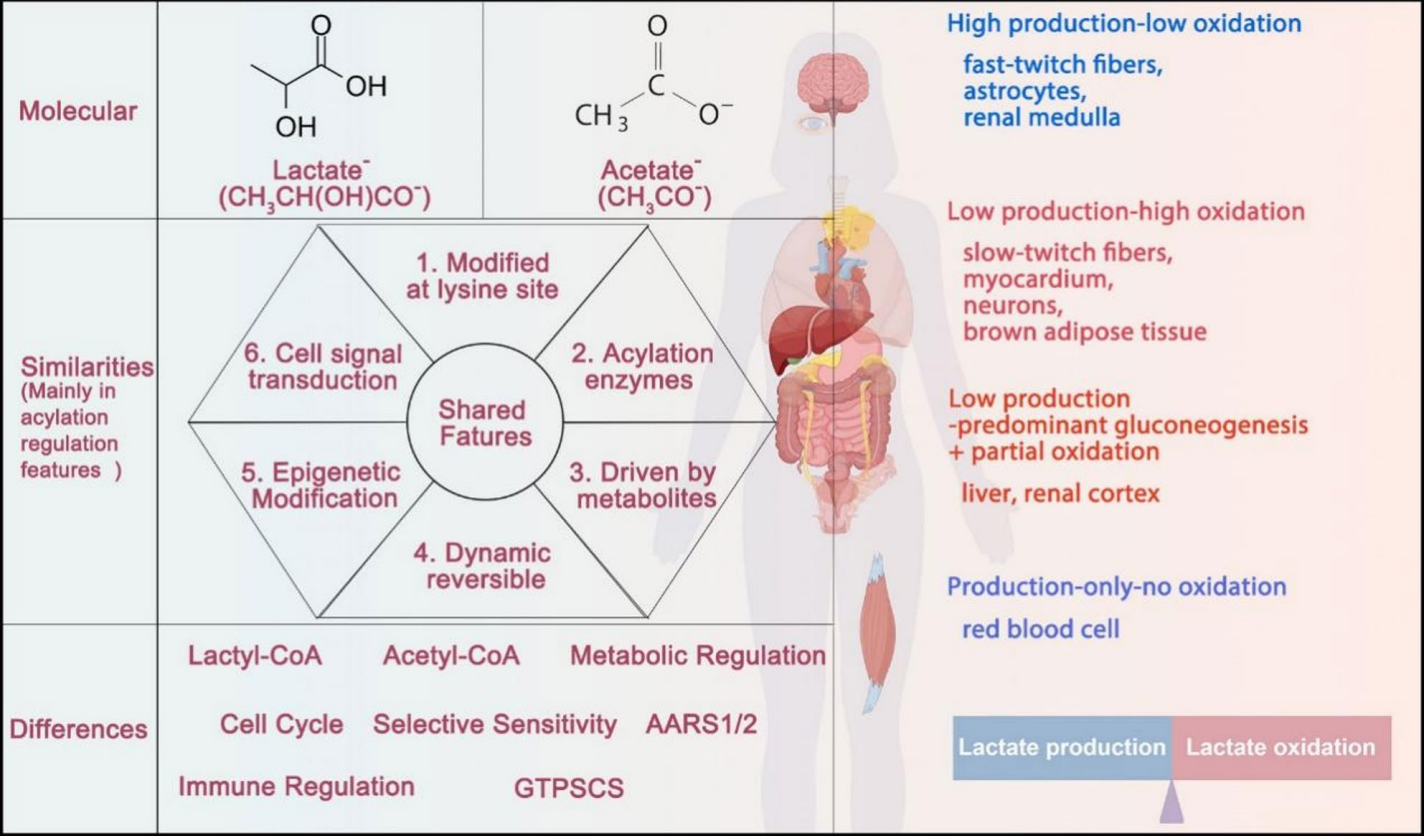
Overview of the Differences and Distinctions between Lactylation and Acetylation. Both lactylation and acetylation are types of acylation modification. They exhibit consistent acylation characteristics. However, differences exist in their substrates (lactate/lactyl - CoA for lactylation, acetate/acetyl - CoA for acetylation), regulatory modes (such as AARS1/AARS2, GTPSCS for lactylation, with specific regulatory factors also existing for acetylation), and functions. AARS: Alanyl-tRNA Synthetase; GTPSCS: GTP-specific succinyl-CoA synthetase. The figure was drawn using Figdraw (https://www.figdraw.com)

Lactylation and acetylation, as differential modifications of proteins caused by metabolism, result in same or different gene regulations and signal transductions. The high similarity between lactylation and acetylation poses obstacles to their verification [16]. Since the role of acetylation in PTMs has been reviewed in detail in previous reports [10], this review will mainly from the perspective of lactylation, systematically discuss the consistencies and differences in regulation and function between lactylation and acetylation, and summarize the current methods for verification of their function (Fig. 1). Through the summary of the differences in the regulatory functions of proteins by lactylation and acetylation, we can better understand lactylation and acetylation from a macroscopic level, which will enrich our cognition of the related modification and disease treatment.

## Lactate and acetyl CoA, the diversion of metabolic flux

**Fig. 2:**
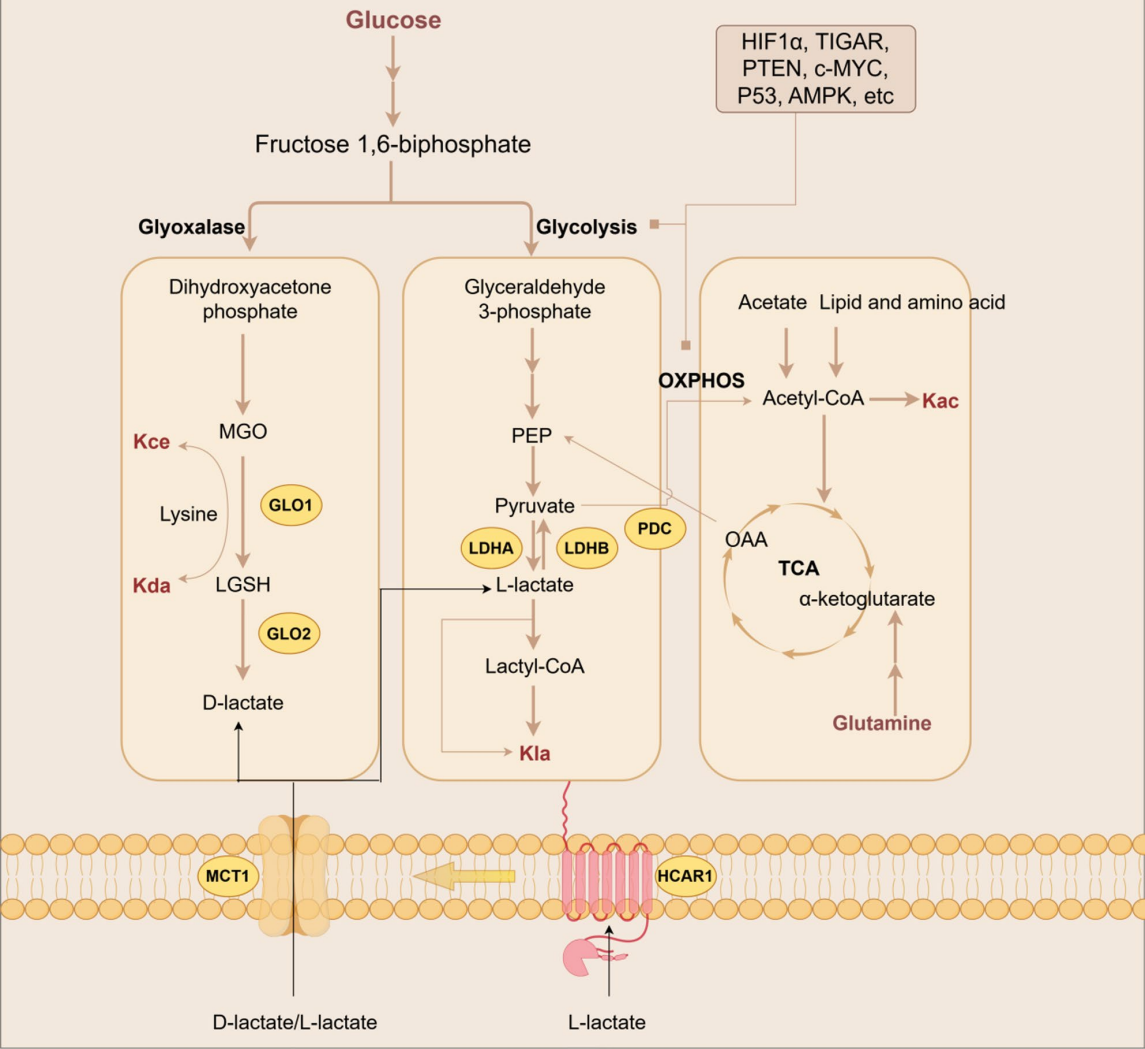
Metabolism of Lactate and Acetyl-CoA. The production of L-lactate mainly occurs through glycolysis. Moreover, glutamine metabolism and extracellular lactate are also important sources of intracellular lactate, supplying substrates for lysine lactylation (Kla). D-lactate is present in relatively low amounts in normal human cells and is predominantly produced by bacteria. In human cells, D-lactate can be generated via the glyoxalase pathway. The intermediate products are methylglyoxal (MGO) and S-lactoylglutathione (LGSH), which respectively serve as substrates for N-ε-(carboxyethyl)-lysine (Kce) and D-lactylation-lysine (Kdla). Acetyl-CoA is the hub of cellular metabolism. It can be produced from the metabolism of glucose, lipids, and amino acids, providing a substrate for lysine acetylation (Kac). GLO: Glyoxalase; MCT: Monocarboxylate transporter; HCAR1: Hydroxycarboxylic acid receptor 1; LDH: Lactate dehydrogenase; PDC: Pyruvate dehydrogenase complex. The figure was drawn using Figdraw

Lactate and acetyl CoA are the substrates of lactylation and acetylation. The main source of endogenous lactate in cells mainly comes from glucose and glutamine, while exogenous lactate is also an important source of lactate within cells [17, 18] (Fig. 2). Acetyl-CoA primarily originates from three major metabolisms: glucose, lipid and amino acids [10]. Due to the impermeability of the membrane, mitochondrial and non-mitochondrial acetyl-CoA pools are produced independently. Mitochondrial acetyl-CoA can be produced by the pyruvate dehydrogenase complex (PDC), fatty acid β-oxidation or amino acid metabolism. The non-mitochondrial acetyl-CoA pool is produced by ATP-citrate lyase, short-chain family member 2 of acetyl-CoA synthetase (ACSS2) and PDC in the cytoplasm and nucleus [19]. Acetyl-CoA diffuse freely between the cytoplasm and the nucleus through nuclear pores [20].

Compared with normal cells, tumor cells and immune cells exhibit a stronger glycolytic capacity, even in the presence of oxygen, called the Warburg effect [21]. Glycolysis and oxidative phosphorylation (OXPHOS) each have their advantages in terms of metabolism. In terms of energy production, OXPHOS is more energy-efficient, generating 30 or 32 molecules of ATP per molecule of glucose [22, 23]. While glycolysis only generates only 2 molecules of ATP per molecule of glucose, but more rapidly. Increased glucose flux also produces other molecules, such as nucleic acids or NAD(P)H [24]. Tumor cells need glycolysis for proliferation and malignancy, while immune cells use it for exerting immune functions [21]. The upstream regulatory genes of glycolysis and the tricarboxylic acid cycle (TCA) are the first factors determining pan-lactylation and pan-acetylation in cells. Protein lactylation is more sensitive than acetylation, allowing lactylation substrates to more readily occupy lysine modification sites than acetyl-CoA [15, 16]. Therefore, the first difference between lactylation and acetylation is the regulation of metabolism.

The selective preferences for glycolysis versus oxidative phosphorylation among different tissues directly determine heterogeneous lactate production/oxidation rates and distinct lactate/acetyl-CoA concentrations. This metabolic trait is consistent across cellular and tissue levels, universally driven by tissue functional specialization. Specifically, tissue lactate metabolism is classified into five phenotypes based on directional tendencies of lactate production and oxidation: (1) High production-low oxidation (fast-twitch fibers, astrocytes, renal medulla), supporting rapid energy for high-intensity exercise, neuronal energy supply, and renal medullary osmotic gradient formation [25–27]; (2) Low production-high oxidation (slow-twitch fibers, myocardium, neurons, brown adipose tissue), facilitating sustained energy, circulating lactate clearance, non-shivering thermogenesis, and synaptic homeostasis [26, 28, 29]; (3) Low production-predominant gluconeogenesis + partial oxidation (liver, renal cortex), as core lactate-clearing organs (≈ 75% of circulating lactate) sustaining blood glucose and acid-base balance via gluconeogenesis [27, 30]; (4) Production-only-no oxidation (red blood cells), relying on glycolytic lactate production for structural and functional maintenance [31]; (5) Low production-low oxidation (white adipose tissue), regulating lipolysis via GPR81 signaling and participating in systemic metabolism [32]. This metabolic selectivity arises from the synergy of tissue functional specialization and optimized energy allocation, with tissue microenvironmental heterogeneity and lactate shuttle (distinct from intra-microenvironmental cells) ensuring inter-tissue metabolic homeostasis. Tissue-specific metabolic differences confer a unique context for lactylation and acetylation, driving their selective preferences in diseases. In summary, investigating the physiological and pathological mechanisms of PTMs requires close integration with tissue-specific metabolic backgrounds for comprehensive insight (Fig. 1).

Numerous transcriptional elements are involved in glycolysis regulation, such as HIF1-α, TIGAR, cMyc, KRAS, p53, PTEN, etc [13, 24]. HIF1-α is a hypoxia sensor, can also be activated by other factors under normoxia, and regulates glycolysis, lactate transport, and the transcription of HK2, PDK1, GLUT1, and MCT4. The persistent activation of oncogenic proteins and the inactivation of tumor suppressor can jointly regulate the transcription of key glycolytic proteins, such as HK2, LDHA, PKM2, ENO1, GLUT1, PFKFB3 and PFK2 [24]. In the case of nutrient deficiency, AMPK phosphorylation will down-regulate the glycolysis flux, allowing more glucose metabolism to enter the TCA. It is noted that lactate is also a source of acetyl-CoA induced by LDHB [33]. In glucose metabolism, LDHA, PDH and PDKs are key diversion points, directing glucose metabolism toward lactate or acetyl-CoA [24, 33]. Notably, genes undergo transcription and translation to produce functional proteins, regulated by complex mechanisms. Briefly, genetic alterations do not necessarily alter enzyme function or final metabolites. Crucially, metabolites exhibit a redundancy effect and can in turn act on gene networks (LDHA, PDH, PDK1), leading to misleading information. Thus, metabolic research requires experimental evidence from final metabolite detection, but this does not imply that genetic testing is meaningless for metabolic studies—indirect genetic evidence can provide directional guidance for such research.

Oncogenic driver genes serve as upstream regulators of metabolic pathways. Although their regulatory mechanism is indirect, targeting these genes may hold greater clinical significance than focusing on individual metabolic enzymes or modification regulators [34]. Oncogenic RAS, particularly KRAS, orchestrates the dynamic equilibrium between lactylation and acetylation through remodeling tumor metabolic circuitry [35–37]. RAS activation fuels aerobic glycolysis, fostering lactate accumulation and lactyl-CoA biosynthesis, which supplies abundant substrates for lactylation of histones and non-histone proteins (e.g., AARS1/2 substrates). Concurrently, it modulates acetyl-CoA bioavailability via regulating fatty acid synthesis and tricarboxylic acid (TCA) cycle reprogramming [36]. In KRAS inhibitor-resistant models, sustained RAS activation aggravates the lactate/acetyl-CoA ratio imbalance, culminating in metabolism-dependent post-translational modification (PTM) dysregulation [38]. Furthermore, RAS-mediated metabolic reprogramming elicits distinct responses of the two modifications: a high-lactate microenvironment augments histone lactylation of oncogenes to facilitate transcriptional activation, whereas non-histone lactylation reinforces metabolic adaptation. Fluctuations in acetyl-CoA and lactyl-CoA levels govern acetylation of cell cycle- and apoptosis-associated genes, establishing synergistic or antagonistic crosstalk with lactylation (e.g., lactylation can substitute for partial acetylation sites under metabolic stress). This metabolism-PTM regulatory axis ultimately propels tumor proliferation, invasion, and therapeutic resistance [37, 39]. In KRAS-resistant tumors, lactate accumulation remodels chromatin and activates resistance-related genes through enhanced lactylation, while suppressing acetylation-mediated growth-inhibitory pathways, thereby establishing a PTM regulatory barrier. Thus, targeting key oncogenes may yield superior therapeutic outcomes for tumor treatment compared with targeting individual metabolic pathways, and targeting both lactylation and acetylation constitutes a promising therapeutic strategy for tumors harboring oncogenic mutations (e.g., KRAS mutations).

D-lactate has an independent metabolic mode. In eukaryotes, there are two forms of D-lactate metabolism. One is the formation of D-lactate through the methylglyoxal pathway (the methylglyoxal pathway is a branch of glycolysis that converts glucose to methylglyoxal and then to D-lactate) [40], and D-lactate can also be produced by the decomposition of glucose by intestinal microorganisms (such as lactobacillus) and absorbed into the blood through the intestine [41]. Past clinical studies have found that abnormal D-lactic acid metabolism is closely related to the occurrence and development of various diseases, such as intestinal diseases, brain diseases, tumors, and other diseases [42–44]. This suggests that D-lactylation, a modification caused by this trace metabolite, may also play an important role in various diseases and has different regulatory mechanisms and functions.

## The regulation of lactylation and acetylation

### Regulatory protein of acylation

**Fig. 3:**
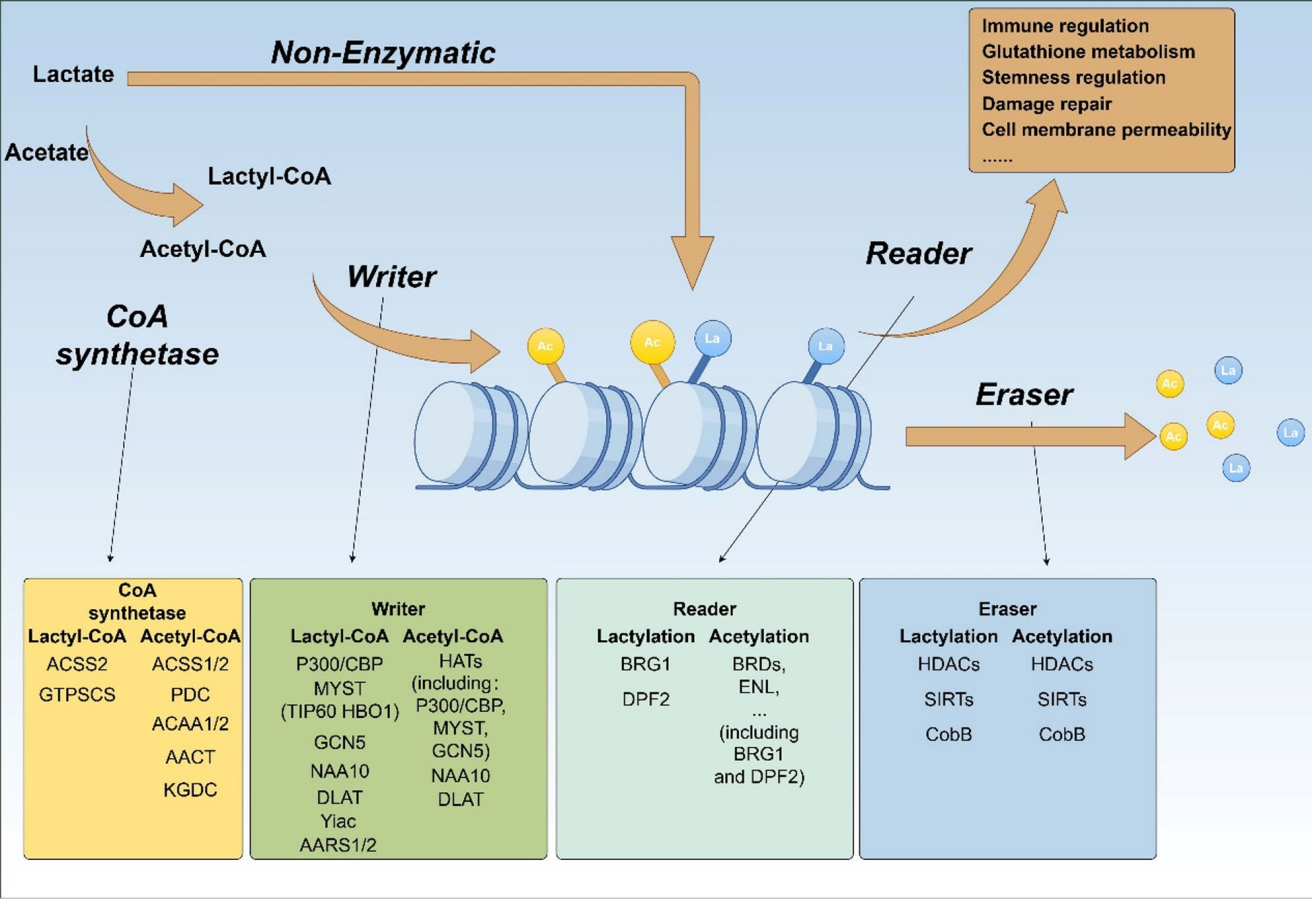
Regulatory Proteins of Lactylation and Acetylation. Both lactylation and acetylation belong to acylation modifications, which are regulated by acyl - CoA synthetases, as well as the so-called “writers”, “readers”, and “erasers”. However, there are also some non-classical regulatory pathways. The figure was drawn using Figdraw

The regulatory mechanisms of lactylation and acetylation are largely similar but also have some differences (Fig. 3; Table 1). Lactylation and acetylation both occur at lysine sites and change the protein conformation by eliminating the anions or pocket occupation [10]. Additionally, both lactylation and acetylation occur through non-enzymatic acylation, and the two systems are different. In enzymatic lysine acetylation modification, lysine is deprotonated by lysine acetyltransferases (KATs) active sites and undergoes acetylation in neutral or acidic environments [45]. While in an alkaline intracellular condition, the deprotonated form of lysine increases, increasing the occurrence of non-enzymatic acetylation reactions [46]. Lactylation also has non-enzymatic acylation reaction form and can occur independently of the conversion of lactate to lactyl-CoA [15]. As mentioned above, lactylation is more sensitive than acetylation. This is because the carboxylic acid groups of these acyl-CoAs induce intramolecular nucleophilic attacks on CoA thioester bonds, forming reactive cyclic anhydrides that efficiently modify protrins non-enzymatically [47].

**Table 1:** Lactylation - regulating enzymes and their roles in acetylation

Protein	Classification	Mechanism	Subcellular localization	Acetylation regulatory protein? (YES/NO)	Ref.
**Lactoyl-CoA synthetase**					
ACSS2	Acyl-CoA Synthetase	Catalyze the generation of acetyl - CoA or lactyl - CoA	Cytoplasm, Nucleus	Y	[48]
GTPSCS	Succinyl-CoA synthetase	Generate lactyl-CoA using lactate, without affecting succinylation	Nucleus (translocates from cytoplasm)	Not Known	[49]
***Writer***					
AARS 1/2	Non-classical lactylation regulatory proteins	Use lactate as a substrate, consume ATP to form Lactate - AMP, and then modify the lysine sites of proteins	AARS1: cytoplasm, mitochondria, extracellular space; AARS2: mainly in mitochondria	N	[14, 15, 50]
P300/CBP	KATs Family	Catalyze the lactylation modification of target proteins, affecting chromatin structure and gene expression	Nucleus	Y	[2]
MYST (HBO1, GCN5, TIP60)	KATs Family	Catalyze the lactylation modification of target proteins, affecting chromatin structure and gene expression	Nucleus	Y	[51–53]
NAA10	N - acetyltransferase	As a catalytic subunit of the NatA complex, transfer lactyl groups to target proteins	Mainly in cytoplasm, also in nucleus	Y	[54]
DLAT	dihydrolipoamide S-acetyltransferase			Y	[49]
Yiac	N - acetyltransferase	Unknown	Cytoplasm (assumed based on general bacterial protein localization)	N	[55]
***Reader***					
BRG1	Chromatin modification recognition proteins	Recognize and bind to lactylated histones, mediating downstream transcriptional changes	Nucleus	Y	[56]
DPF2	Chromatin modification recognition proteins	Recognize and bind to lactylated histones, mediating downstream transcriptional changes	Nucleus	Y	[57]
***Eraser***					
HDAC1-3, 6	Histone Deacetylases (Zn²⁺ - dependent)	Catalyze the removal of acetyl or lactyl groups from histones or non - histone proteins	Class I and Class IV: nucleus; Class IIb: cytoplasm; Class IIa: mainly in nucleus, translocate to cytoplasm upon signal activation	Y	[58–63]
SIRT1-3	Deacetylases (NAD⁺ - dependent)	Catalyze deacetylation and delactylation reactions using NAD⁺as a co-factor	SIRT1 and SIRT6: nucleus; SIRT7: nucleolus; SIRT2: cytoplasm; SIRT3, SIRT4, and SIRT5: mitochondria	Y	[58–63]
CobB	Sirtuin-like deacylase	unknown	Cytoplasm (in bacteria)	Y	[55]

This table summarizes core lactylation regulators (including Lactoyl-CoA synthetase, writers, readers, and erasers), along with their functional characteristics and whether they participate in acetylation regulation

In the traditional enzymatic acylation reaction, acyltransferases and deacylases form a single system. Here, we mainly provide a brief explanation using the most deeply studied acetylation regulatory proteins as an example. The exact number of true KATs in the human proteome remains unclear. Among the reported KATs, the majority are classified into three families: GCN5, P300, and MYST [10]. The remaining KATs, including histone acetyltransferase 1 (HAT1; also known as KAT1), α-tubulin N-acetyltransferase 1 (TAT1; also known as ATAT1), establishment of cohesion 1 homolog 1 (ESCO1), and ESCO2 [10]. Due to the different subcellular localizations of these KATs, the targeted proteins also differ. Different KATs induce variations in acyl modification sites, but some also overlap. As the core subunit responsible for acetyl group transfer in the pyruvate dehydrogenase complex (PDC), dihydrolipoamide S-acetyltransferase (DLAT) also possesses independent protein acetyltransferase activity. It can target a variety of substrates to regulate their acetylation and functions, thereby affecting metabolism, signaling pathways, and disease progression. In recent studies, DLAT has been identified to act as a lysine lactyltransferase as well, and it exerts a pro-pathogenic effect on myocardial ischemia-reperfusion injury (MI/R) [51, 52]. The lysine deacetylases (KDACs) encoded by the human genome can be classified into two major categories: Zn2+-dependent HDACs and NAD+-dependent sirtuin deacetylases [10]. The Zn2+-dependent HDACs possess a highly conserved deacetylase domain. The sirtuin protein family is also structurally conserved, and they typically contain a catalytic core region that binds to nicotinamide adenine dinucleotide (NAD+). In terms of KATs, except for TAT1, typical KATs are mainly located in the nucleus and are responsible for acetylating histones and nuclear non-histone proteins. For lysine deacetylases (KDACs), Zn²⁺-dependent histone deacetylases are classified into Class I, Class IIa, Class IIb, and Class IV based on sequence similarity. Among them, Class I and Class IV KDACs are located in the nucleus, Class IIb KDACs exist in the cytoplasm, and signal-responsive Class IIa KDACs are mainly in the nucleus and will transfer to the cytoplasm upon signal activation. Sirtuin deacetylases, known as Class III KDACs, are distributed in different cellular compartments, such as the nucleus (SIRT1 and SIRT6), nucleolus (SIRT7), cytoplasm (SIRT2), and mitochondria (SIRT3, SIRT4, and SIRT5). It is noted that nearly half of the deacetylases have weak deacetylase activity or no deacetylase activity and target other acylation modifications.

### High similarity in regulatory proteins of lactylation and acetylation

The enzymatic acylation form of lactylation reaction does not deviate from the category of acylation reactions. Correspondingly, the KATs that lactylation regulation depends on are the same as those of acetylation, with regional selectivity of subcellular structures [10]. For example, KATs that play a regulatory role in the nucleus, such as P300/CBP, MYST (TIP60, HBO1), and GCN5, have all been found to play a regulatory role in nuclear protein lactylation [2, 53, 54, 64]. Furthermore, NAA10, as the catalytic subunit of the NatA (N-acetyltransferase A) complex, and ACAT2, Acetyl-CoA Acetyltransferase 2, has also been found to participate in the regulation of lactylation and acetylation modification [35, 65, 66]. The generation of acetyl- or lactyl-CoA relies on the acyl coenzyme A synthetase ACSS2, which can utilize lactate as a substrate to produce lactyl-CoA, as confirmed by Zhu et al. [48]. GTP-specific succinyl-CoA synthetase (GTPSCS) has also been identified as the synthase of intranuclear lactyl-CoA, yet its effect on acetyl-CoA remains unclear [49]. Despite sharing substantial structural similarity with succinyl-CoA synthetase, GTPSCS stands out as a definitive example of functional divergence among “writers” as it translocates to the nucleus, interacts with p300, and exclusively enhances histone lactylation—with no impact on succinylation. This process depends on the nuclear localization signal (NLS) on the G1 subunit of GTPSCS and the acetylation modification at the K73 site of the G2 subunit, the latter mediating its binding to p300 [49].

Recently, a series of studies based on crispr/cas9 libraries have reported the role of AARS1/2 in the regulation of protein lactylation [14, 15, 50]. AARS1/2 is mainly located in the cytoplasm. It is worth noting that the substrate of lactylation mediated by AARS is not lactyl CoA but lactate. After consuming one molecule ATP, lactate combines with AMP to form Lactyl-AMP, and then still under the action of AARS1/2, it binds to the lysine site of the protein and releases AMP, forming lysine lactylation modification [15]. AARS is highly conserved throughout evolution and plays a key role in protein synthesis. Its main function is to catalyze the binding of alanine to the corresponding tRNA (transfer ribonucleic acid), and this process is highly specific [67]. Specifically, AARS can recognize alanine and its corresponding tRNA (tRNA Ala). But interestingly, the sensitivity of AARS to lactate is much higher than alanine and acetyl CoA [14]. This means that the lactylation induced by AARS is specific. However, it is unclear whether other metabolites can undergo corresponding metabolic modifications through AARS. Simultaneously, significant differences exist in the data of lactylome mediated by AARS1 and AARS2. This difference may be related to the subcellular localization of AARS1 and AARS2 [15]. AARS1 is located in the cytoplasm, mitochondria, and extracellular regions, while AARS2 is mainly located in mitochondria and is much higher than other subcellular structural regions.

A recent study on lactylation readers identified BRG1 as a potential reader of H3K18la [56], and its interaction with acetylation was reported earlier [68, 69]. A similar situation was discovered in DPF2. By combining multivalent photoaffinity probes and quantitative proteomics methods, the authors identified DPF2 as a candidate target of H3K14la [57], but DPF2 is also a “reader” of acetylation [70]. Some typical acetyllysine readers, including bromodomain-containing proteins, can also recognize other lysine acylations, such as propionylation, butylation, and butyrylation, although the selectivity levels are different [47]. In the study of delactylation, or the Eraser of lactylation, the main reports are still concentrated in the HDAC and SIRT families, and the corresponding reports have no major differences from acetylation [58–63]. Meanwhile, in Escherichia coli, CobB and Yiac have been found to be involved in the erasing and writing of lactylation respectively, yet no such findings have been reported in human cells [55].

### Metabolism and subcellular regulation of Lactyl-/Acetyl-CoA

Lactylation and acetylation depend on the metabolic context of different tissues or cells, and the subcellular distribution of acetyl-CoA, lactyl-CoA, and their regulatory enzymes across organelles in single cells provides a valuable discussion point for their pathway-specific contributions under distinct contexts.

In mitochondria, acetyl-CoA is mainly generated via PDH-catalyzed pyruvate conversion, fatty acid β-oxidation, and amino acid catabolism. Its high concentration dominates mitochondrial protein acetylation and indirectly supplies substrates for cyto-nuclear acetylation via the citrate shuttle. Lactyl-CoA is barely synthesized in mitochondria (weakly catalyzed by ACSS1 and AARS2 from lactate only under pathological conditions) [71, 72], thus contributing minimally to lactylation with only faint modification effects under extreme circumstances.

In the cytoplasm, acetyl-CoA is produced by ATP-citrate lyase (ACLY)-mediated cleavage of mitochondrial-derived citrate and acetyl-CoA synthetase 2 (ACSS2) catalyzed acetate activation [73]. It serves as the core substrate for cyto-nuclear acetylation and competes with lactyl-CoA for modifying enzymes. Lactyl-CoA, primarily synthesized by ACSS2/ACSS1 and AARS1/2 via lactyl-CoA ligation, is the major substrate for lactylation [15]. Its level dictates the intensity of cyto-nuclear lactylation and can substitute for partial acetylation functions under pathological conditions.

In conclusion, lactylation involves both acyl modification and non-acyl regulatory mechanisms. At the acyl modification level, lactylation and acetylation share the same regulatory machinery, making it difficult to differentiate. In the non-acyl regulatory mechanism of lactylation, the regulatory role of AARS on lactylation is different from that of acetylation. Additionally, the subcellular localization and regional effects of regulatory enzymes merit emphasis.The high similarity between the regulatory mechanisms of acetylation and lactylation poses significant challenges for lactylation research and necessitates a more cautious interpretation of past acetylation studies.

## Lactylation function: contrasted with acetylation

**Fig. 4:**
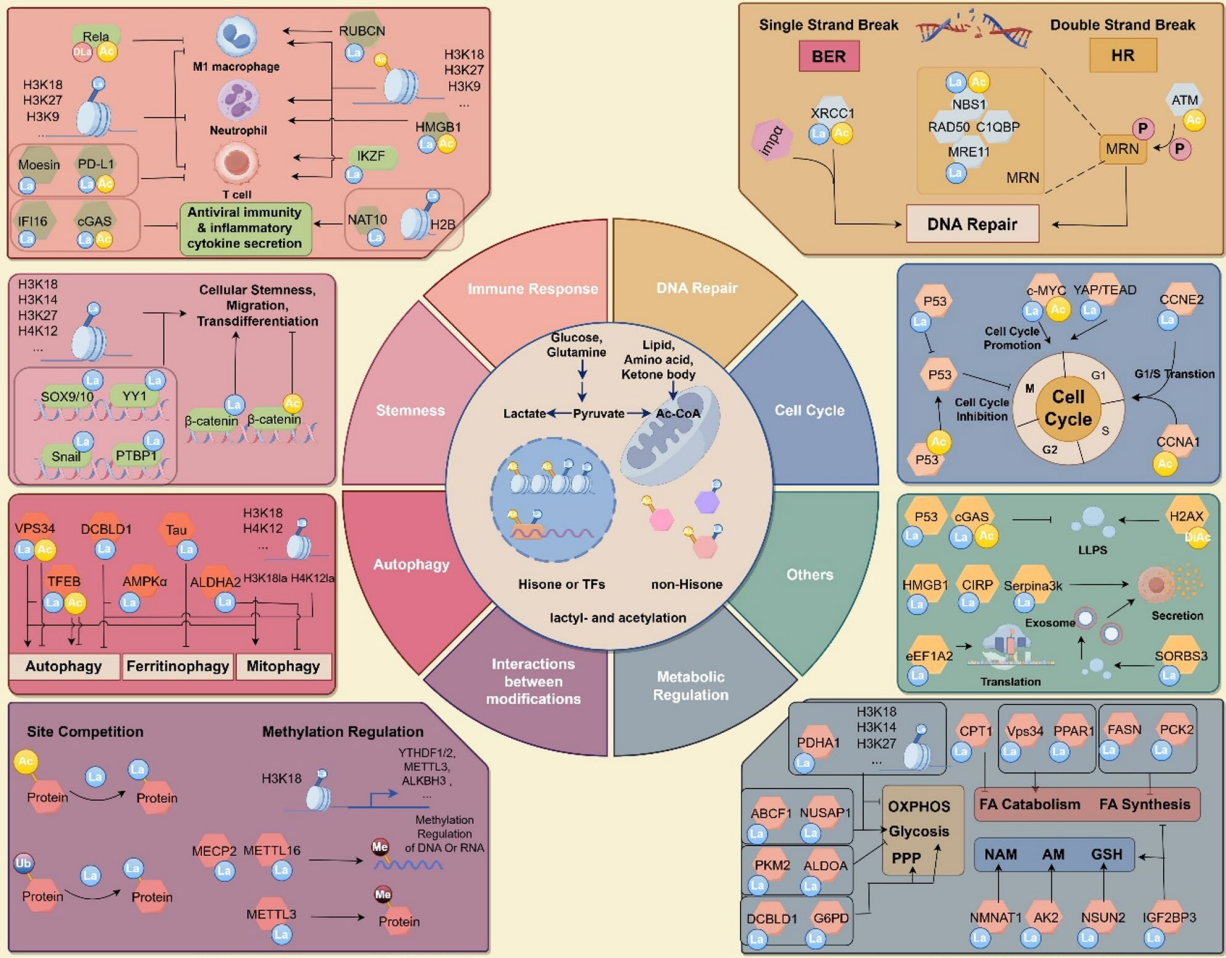
Functions of Lactylation. Lactylation occurs on a variety of proteins and plays a crucial role in regulating multiple phenotypes. Here, we roughly categorize lactylated proteins into eight groups: immune regulation, stemness, DNA repair, autophagy, cell cycle, metabolic regulation, interaction between modifications, and other functions. Although the role of acetylation is partially indicated, the focus is mainly on the functions of lactylation. LA: lactylation; AC: acetylation; Ub: ubiquitination; BER: Base Excision Repair; HR: Homologous Recombination Repair; LLPS: Liquid - Liquid Phase Separation; FA: Fatty acid; PPP: Pentose Phosphate Pathway; NAM: Nicotinamide metabolism; AM: Adenylate metabolism GSH: Glutathione. The figure was drawn using Figdraw

Lactylation and acetylation, as two significant PTMs, have been found to be involved in various cellular processes. Although their regulatory mechanisms are similar, they differ in their functional outcomes. The similarities and differences between lactylation and acetylation will be further discussed in the following sections (Fig. 4; Table 2).

**Table 2:** The function of lactylated proteins and its consistency with acetylation. QA: any reports on acetylation? (YES/NO) QB: is the acetylation effect consistent or opposite? (CONSISTANT/OPPOSITEBIDIRECTIONAL EFFECT/NOT KNOWN)

Protein	Site	Model	Function	Regulatory protein	Ref.	QA	QB
**Gene transcription**							
Immune							
H3	K9/14/18la	Macrophage, T cells, GBM and MPE	Suppress the function of M1-type macrophages, T cells, NKT, and neutrophils, induce the formation of M2-type macrophages and Treg.	ACSS2, CBP/P300 and HBO1	[2, 51, 74–76]	Y	O
H2B	K16la	Swine fever virus (CSFV) infection	Activate the NF-κB signaling pathway inhibit the CSFV replication.		[77]	Y	O
IKZF1	K164la	EAU	Promote the transcription of TH17 differentiation genes		[7]	N	N
Stemness and Angiogenesis							
H3	K14/18la	GBM, GC, embryonic tissue and Diabetic nephropathy	Promote stemness maintenance and stem cell proliferation	GTPSCS, P300	[2, 66, 78–82]	Y	C
H2B	K58la	HCC	Enhance senescence resistance and stemness.	CBP/p300	[83]	Y	C
Snail1	Kla	PC and MI	Promote the EMT process		[84, 85]	Y	C
β-catenin	Kla	CRC	Promote the Wnt signaling pathway		[86, 87]	Y	C
PTBP1	K436la	GSC	Promote stemness maintenance		[87, 88]	Y	N
SOX9/10	Kla	VSMC, NSCLC	Affect the glycolysis, cellular stemness, migration, and transdifferentiation		[88, 89, 159]	Y	N
YY1	Kla	Microglia and EAU	Promote angiogenesis		[90, 161]	Y	B
Metabolism							
H3	K12/14/18la	HCC, PDAC and HSC	Promote glycosis	CBP/p300	[66, 91, 163–165]	Y	C
H4	K12la	Fibroblast	Promote collagen synthesis	KAT5-KAT8, HDAC3	[92, 166]	Y	C
Methylation							
H3	K18la	Melanoma, Sepsis-ALI, PML, IPF	increase the expression of YTHDF1/2, METTL3, ALKBH3	CBP/p300	[93–97]	Y	C
***Immune regulatory***							
RELA	K310dla	Macrophage	Inhibits immune activation of macrophage		[5]	Y	C
PD-L1	K270/271la	CRC	Increase stability of PD-L1, Inhibit cytotoxic T cell-mediated antitumor responses	GCN5	[97, 175]	Y	C
RUBCN	K33la	Macrophage, Bacterial infection	Promote anti-bacterial immune responses		[98, 103]	N	N
cGAS	K131la(H), K156la(M)	HSV1 infection	Hinder the LLPS of cGAS and inhibit the binding of cGAS to DNA	AARS2	[15]	Y	C
HMGB1	Kla	Hepatocytes, Sepsis and AKI	Promote the secretion and NETs		[99–102]	Y	C
Moesin	K72la	T cell	Promoting the generation of Treg		[98, 102]	N	N
IFI16	K90la	HCMV and HSV1 infection	Prevent IFI16-related anti-virus response	AARS1	[50]	Y	O
NAT10	K290la	KSHV infection	Promote the reactivation of KSHV	ATAT1	[103, 177]	Y	N
S100A9	K26	MI/R	promoting neutrophil migration and cardiac recruitment	DLAT	[49, 52]	Y	N
***Cell Cycle***							
CCNE2	K348la	HCC	Induce the transformation from the G1 to S phase	SIRT3	[104, 179]	N	N
P53	K120/139la	BC	Impedes LLPS and transcriptional activation of P53		[14]	Y	B
c-myc	Kla	Kidney IRI	Promote the stability and function of c-MYC		[105, 106]	Y	C
YAP/TEAD	K9la (YAP), K108la (TEAD)	GC	Promote the proliferation of GC cells	AARS1	[106, 185]	Y	C
***DNA Repair***							
XRCC1	K247la	GBM	Enhance the mode of BER of DNA		[107, 186]	Y	O
MRE11	K673la	CRC	Enhance the mode of HR of DNA	CBP/p300	[108, 109]	N	N
NBS1	K388la	pan Cancers	Enhance the mode of HR of DNA	TIP60, HDAC3	[108, 109]	N	N
RAD51	K73la	Ovarian Cancer	Enhance the mode of HR of DNA	GCN5	[64, 110]	Y	B
***Metabolic regulation***							
ABCF1	K430la	HCC	Activate the KDM3A-H3K9me2-HIF1A axis	P300, HDAC1,3	[111]	N	N
NUSAP1	Kla	PDAC	Inhibit NUSAP1 degradation, binding to c-Myc and HIF-1α		[112]	N	N
PKM2	K62la	Macrophage	Inhibit Warburg effect		[113, 191]	Y	O
ALDOA	K230,322, 147la	HCC, E. Coli and Mammalian cells	Inhibit the enzymatic activity of ALDOA and glycolysis		[8, 114, 115]	Y	C
DCBLD1	K172la	Cervical cancer	Activate PPP pathway		[115, 116]	N	N
G6PD	K45la	Cervical cancer	Activate PPP pathway		[116, 117]	Y	B
CPT1	K457/8la	muscle cell	Inactivate enzyme and inhibit fatty acid oxidation	AARS2, SIRT3	[118, 198]	Y	C
PDHA1	K336la	muscle cell	Inactivate enzyme and inhibit OXPHOS from pyruvate	AARS2, SIRT3	[118, 198]	Y	B
Fis1	K20la	AKI	Promote mitochondrial fission, production of mtROS		[118, 119]	Y	C
Vps34	K356,781la	GC, Lung Cacncer	Increases Vps34 lipid kinase activity	KAT5/TIP60	[119, 120]	Y	O
PPAR1	K498,505,506,508,518,521,524la	293T	Increase ADP-ribosylation activity		[55]	Y	N
FASN	K673la	HCC	Inhibit the activity of FASN		[199, 121]	Y	B
PCK2	K100la	Hepatic IRI	Reduce the β-oxidation, and enhance oxidative phosphorylation	KAT8	[119, 122]	N	N
NMNAT1	K128la	PDAC	Enhance the NAD+ salvage pathway	P300, SIRT1	[123, 129]	N	N
AK2	K28la	HCC	Promotes enzymatic activity		[6]	N	N
IGF2BP3	K76la	HCC	IGF2BP3la-PCK2-SAM-m6A elevates PCK2 and NRF2 levels		[123, 124]	N	N
NSUN2	K508la	HCC	Induce GSH synthesis	NAA10	[54]	N	N
GCLM	K34la?	PDAC	Promoting GSH synthesis	ACAT2?	[35]	N	N
HDAC1	K412la, K438la	ESCs	Enhances its deacetylase activity		[59, 125]	Y	O
***Ubiquitination***,*** Methylation and Autophagy***							
NEDD4	K33la	Macrophage	Inhibit the binding of NEDD4 to other proteins		[126, 205]	Y	C
METTL3/16	K27 (M3), K229la (M6)	TNBC, ICH and GC	Enhance the methylation of mRNA or proteins		[60, 127, 128]	Y	B
MECP2	K271la	ASCVD	Enhance protein function and methylation recognition		[129, 201]	Y	N
TFEB	K91la	Hela	Increased lysosomal activity and autophagic flux.		[126, 130]	Y	B
Tau	K667la	AD	Inhibit ferritinophagy		[131, 202]	Y	N
AMPKα	Kla	Intervertebral Disc	Inhibit autophage	P300	[132, 190]	Y	N
ALDH2	K52la	AKI	Inhibit mitophagy	SIRT3	[133, 141]	Y	C
***Molecules secretion***							
SORBS3	K479la	Muscle cell	Induced LLPS prompts FBXO2 sorting into small extracellular vesicles		[134, 167]	N	N
HMGB1	Kla	Hepatocytes, Sepsis and AKI	Promote the secretion and NETs		[99, 100]	Y	C
CIRP	Kla	ALI (macrophages and PVEC)	lead to the release of CIRP		[135, 209]	Y	N
Serpina3k	K351la	Cardiac IRI	enhance protein stability and secretion		[136, 206]	N	N
***Others***							
Twist1	K150la	Skin Fibrosis	Promotes the nuclear translocation of Twist1, inducing the fibrotic phenotype		[137, 142]	Y	C
eEF1A2	K408la	CRC	Increase in translation elongation rate and enhanced protein synthesis	KAT8	[138, 140]	Y	N
α-MHC	K1897la	Heart Failure	promote interaction between α-MHC and Titin, thereby alleviating heart failure	P300, SIRT1	[136, 206]	N	N
ARF1	K73la	Astrocyte	Reduce mitochondrial transfer to damaged neurons and exacerbates IRI		[139, 211]	N	N
APP	K612la	AD	Accelerate the endosomal - lysosomal degradation pathway of APP		[140, 210]	Y	C
LCP1	Kla	Ischemic Stroke	Enhance protein stability and progression of ischemic stroke		[136, 141]	N	N
α-tubulin	K40la	Hippocampal Neurons	Enhance microtubule dynamics and promote neurite outgrowth	HDAC6	[58]	Y	N
Egr1	K364la	S-ALI	Promote the interaction with importin - α, facilitating its nuclear localization and contributing to S-ALI related glycocalyx degradation and ALI	P300	[135, 142]	N	N

This table summarizes the functions of lactylated proteins, as well as the consistency between their functional characteristics and the acetylation functions of the corresponding proteins. The consistency categories are classified into four types: consistent (C), opposite (O), bidirectional effect (B), and unknown (N)

### Gene transcription

In the initial study of histone lactylation, Zhang et al. discovered that lactylation can act as a “lactylation clock” in macrophages [2]. In lipopolysaccharide (LPS)-activated M1 macrophages, there is a competitive relationship between P300-mediated lactylation and acetylation. As the lactylation level increases, macrophages gradually transform to the M2 type. Similarly, Irisari-Caro et al. found that BCAP, a B-cell adaptor protein, promotes histone lactylation by regulating glycolysis, driving the transformation of inflammatory macrophages to reparative ones [74]. Meanwhile, Dichtl et al. further investigated whether Kla is the result or cause of M2 macrophage activation and verifying the absence of Kla formation in macrophages that are unable to produce nitric oxide or have defects in different death pathways [75]. This brings a new perspective on the role of histone lactylation in immune regulation. Additionally, modification regulatory enzymes exhibit substantial functional divergence across different models. For instance, the canonical acyltransferase P300—a classic acetyltransferase and the first identified lactyl-CoA transferase—can both promote inflammatory responses and suppress the sustained activation of inflammatory pathways in distinct models [76]. The “clock” mechanism prevents sustained overactivation of inflammatory responses, a notion indirectly supported by P300-mediated metabolic regulation. Numerous studies have demonstrated that P300 activates multiple glycolysis-related metabolic enzymes [143], reflecting its critical roles in both immune activation and metabolism—suggesting P300 is not only a key participant but also a driver of the “lactate clock” in immunity.

Histone lactylation has been linked to immune suppression in lung cancer, gastric cancer, prostate cancer, acute lymphoblastic leukemia, colon cancer, bladder cancer, malignant pleural effusion, and inflammatory bowel disease [144]. The expression of arginase-1 regulated by histone lactylation is also required for the suppression of T cells and neutrophils within brain tumor [145–147]. In the CAR-T treatment of glioma, H3K18la upregulated the expression of CD39, CD73, and CCR8, inhibiting the effect of immunotherapy [148]. In tumor-induced malignant pleural effusion (MPE), H3K18la is considered to be associated with the formation of regulatory T cells (Tregs) and the regulation of natural killer T cells (NKT cells) [147, 149]. The immune functions of histone acetylation differ from that of lactylation. In glial cells, H3K27ac promotes the transmission of secondary inflammatory signals [150]. In a preclinical leukemia CAR-T model, the decrease in IDH2-mediated histone acetylation impairs the therapeutic effect of CAR-T [151].

In inflammatory diseases, the K164 lactylation of the transcription factor (TF) IKZF1 promotes TH17 differentiation gene transcription and enhances experimental autoimmune uveitis (EAU) progression [7]. In the course of Classical Swine Fever Virus (CSFV) infection, H2B undergoes lactylation modification [77]. This modification enables the translocation of p65 into the nucleus with the aid of karyopherin alpha 2 (KPNA2), subsequently activating the nuclear factor kappa B (NF-κB) signaling pathway. Histone acetylation, by contrast, promotes the progression of some inflammatory diseases. PHGDH maintains the expression of IL-1β through Toll-like receptor 4 transcriptional activation mediated by H3K9/27 acetylation [152]. In LPS-induced systemic inflammation, myeloid-specific PHGDH knockout mice displayed reduced histone acetylation and alleviated inflammation. H3K18ac promotes the secretion of IL − 1β, IL − 6 and IL − 17 in lipopolysaccharide (LPS) - induced macrophages. Moreover, it is closely associated with the function of CD4 + T cells [153, 154]. The contrasting roles of lactylation and acetylation in the regulation of inflammatory-related genes are interesting. The increase in lactylation serves as a “stop” signal to prevent excessive immune activation.

In the transcriptome regulated by related H3K18la revealed by Zhang et al., the overall transcriptional changes brought by H3K18la also include gene sets related to glutathione metabolism, stem cell characteristics, damage repair, and cell membrane permeability [2]. However, the role of acetylation in cellular stemness or EMT is consistent. In the cellular senescence model, Glis1 activates the glycolysis gene switch [78]. The coordinated histone lactylation and acetylation mediated by Glis1 regulates the cellular reprogramming and pluripotency. In mice, histone lactylation is found to regulate the proliferation of neural stem cells through the MDM-P53 pathway [79]. In diabetic nephropathy and osteoblast differentiation, the histone lactylation is considered related to the Maintenance of pluripotency [80, 81]. In tumor models, histone lactylation has also demonstrated an extensive role in promoting stemness. In gastric cancer and glioblastoma, H3K18la promotes tumor metastasis and stem cell maintenance by activating the AKT-mTOR-CXCL1 axis and generating the MAP4K4/JNK pathway regulated by LINC01127, respectively [49, 82, 83]. In HCC, H2B lactylation promotes tumor senescence resistance and heterogeneity [155].In the gastric cancer model, H3K27 acetylation upregulates LINC00501 to promote metastasis by activating EMT and angiogenesis [156]. The acetylation of histone by HBO1 (KAT7) activates the Wnt/β-catenin signaling pathway and promotes the progression of B-cell acute lymphoblastic leukemia and the dormancy of liver cancer [157, 158]. Lactate regulates MYC expression by enhancing histone acetylation and chromatin accessibility at the MYC locus in a BRD4-dependent manner, thus promoting the proliferation of CSCs and maintaining their stemness [84]. The lactylation of stemness-related transcriptional factors (TFs) is also involved in stemness regulation. Lactylation of the stemness-related TFs, Snail1, PTBP1, β-catenin and SOX9/10 show the induction of stemness in cancer and vascular smooth muscle cells [85–89, 159].

Angiogenesis-related genes often show a consistent trend with stemness-related gene sets, and lactylation and acetylation play similar roles. H3K18la and H3K27ac jointly regulate angiogenesis, and the acetylation of ZEB1 mediates the expression of angiogenesis-related genes [90, 160]. However, in retinal neovascularization, YY1 lactylation in microglia promotes angiogenesis and cancer metastasis by upregulating fibroblast growth factor 2 (FGF2) and FBXO33, while YY1 acetylation inhibits its stability and DNA binding activity [91, 161, 162].

Lactylation and acetylation jointly activate the expression of glycolysis, fibrosis and methylation-related genes to activate the malignant progression of cancer or disease. GTPSCS collaborates with p300 to coordinately regulate histone H3K18la lactylation and the expression of GDF15, promoting the proliferation and radioresistance of gliomas [49]. The ubiquitin E3 ligase NEDD4 was identified activited by H3K14la, mediating PTEN degradation [163]. The downregulation of PTEN forms a positive feedback. In pancreatic ductal adenocarcinoma (PDAC) and hepatic stellate cells (HSC), H3K18la promotes the expression of HK2 and the positive feedback pathway of H3K18la/TTK/LDHA enhances glycolysis, facilitating the proliferation of PDAC and the progression of liver fibrosis [164, 165]. The promoting effect of histone acetylation on glycolysis is similar to that of lactylation, with no obvious difference in tumor models [92]. H4K12la promotes collagen synthesis in fibroblasts by activating TGF-β related pathway [166]. In kidney and Crohn’s diseases, studies have found that H3K18la and H3K9la can activate the transcription of FGFR4 and the EMT pathway respectively, inducing the formation of kidney stones and intestinal fibrosis [134, 167]. Histone lactylation also plays a crucial role in ferroptosis related metabolism. H3K18la-promoted transcription of HECTD2 acts as an E3 ubiquitin ligase for KEAP1 [168]. This promotes KEAP1 protein degradation, releases the NRF2 signaling pathway, and initiates the antioxidant response in HCC cells. H3K14la and H3K18la drives endothelial dysfunction and tumor development by promoting iron metabolism [169, 170]. Acetylation has the similiar effect on iron metabolism [171]. The histone acetyltransferase TIP60 also has an impact on the expression of HAMP, FTH, and FPN [172].

Histone lactylation promotes the transcription of RUBCNL/Pacer, facilitating autophagosome maturation by interacting with BECN1 (beclin 1) [173]. H4K12la -regulated NLRP3 is involved in cigarette-accelerated Alzheimer’s-like pathology through mTOR-regulated autophagy and microglial activation [174]. In the case of traumatic brain injury (TBI) with controlled cortical impact (CCI), histone lactylation upregulates the transcription of PSMD14 [93]. The mitophagy induced by PSMD14 promotes mitochondrial homeostasis, thereby reducing the production of reactive oxygen species. In melanoma, sepsis-associated lung injury (ALI), promyelocytic leukemia protein (PML) and idiopathic pulmonary fibrosis (IPF), histone lactylation induce METTL3 and YTHDF2 expression, promoting N6-methyladenosine (m6A) modification and reading [94–97].

In conclusion, histone lactylation and acetylation exhibit distinct effects on immunity, but their impact on gene expression is largely similar. While it is intuitive that the regulatory effects of epigenetic modifications on genes are different, the underlying mechanisms remain unclear.

### Immune regulatory

The lactylation of programmed death-ligand 1 (PD-L1) mediates immune escape during cytotoxic T cell-mediated antitumor responses, inhibits proteasome degradation induced by PD-L1 ubiquitination, and blocking the PD-1/PD-L1 signaling pathway is able to rejuvenate the function of CD8 + T cells recruited by the -SG diet (restricted serine/glycine diet) in anti-tumor models [175]. PD-L1 acetylation also affects the stability of PD-L1 and its nuclear localization, thereby suppressing the efficacy of tumor immunotherapy [176]. Lactylation may affect certain protein functions by regulating the liquid-liquid phase separation state (LLPS). In recent reports, it was reported that the lactylation (H131, M156) of cGAS can hinder the liquid-liquid phase separation of cGAS and inhibit the binding of cGAS to DNA, thereby affecting the production of downstream type I interferons [15]. Acetylation of cGAS also plays the same role, inhibiting cGAS activity [99]. In macrophages and liver cancer cells, the lactylation or acetylation of HMGB1 may promote the secretion of HMGB1, and this promoting effect may be related to exosomes [100, 101]. While in AKI of mouse, this secretion of HMGB1 induced by lactylation can activate the HMGB1-NETs signaling pathway [102]. In Treg cells, the lactylation of Moesin improves the interaction between Moesin and transforming growth factor β (TGF-β) receptor I, promoting the generation of Tregs [98]. S100a9 lysine 26 lactylation (S100a9K26la) drives the progression of MI/R [52]. Mechanistically, DLAT-mediated lactylation of S100a9 promotes its translocation into the nucleus of neutrophils. In the nucleus, S100a9K26la binds to the promoters of migration-related genes and acts as a co-activator to enhance their transcription, thereby facilitating neutrophil migration and cardiac recruitment.

RUBCN lactylation promotes the binding of RUBCN to Vps34 and the formation of LAP (LC3-associated phagocytosis), and anti-bacterial protective responses. In antiviral protective responses, human cytomegalovirus (HCMV) infection utilizes lactate to induce widespread protein lactylation and promote viral dissemination [103]. K90 lactylation of the viral DNA sensor IFI16 inhibits the recruitment of the DNA-damage-response kinase DNA-PK, preventing IFI16-driven viral gene repression and cytokine induction [50]. However, acetylation of IFI16 leads to the interaction between IFI16 and ASC and the assembly of the inflammasome, consequently resulting in the production of interferon-β. KSHV infection coordinates NAT10 and ATAT1 to enhance NAT10 lactylation, leading to tRNASer-CGA-1-1ac4C modification, ultimately promoting the reactivation of KSHV [177].

Both autocrine or blood-absorbed D-lactic acid accumulation have been found to be associated with the immunosuppressive phenotype of macrophages [178]. Another study revealed the unique D-lactylation process induced by non-enzymatic reactions of S-D-lactic acid glutathione (SLG) in activated innate immune cells [5]. Further studies using lactylation modification omics technology identified a total of 2,255 lactylation modification sites in activated macrophages and clarified the mechanism by which D-lactyl modification of RelA K310 inhibits immune activation and inflammatory signal transduction [5]. Therefore, the GLO2/SLG/D-lactylation regulatory axis is part of the immunometabolic autoregulatory circuit that limits the inflammatory response.

### Cell cycle

Lactate-mediated inhibition of SENP1 can stabilize the SUMOylation of APC4, facilitating the binding of APC/C and UBE2C, which enhances the degradation of cyclin B1 and securin, thereby regulating mitotic exit and detailing the regulation of the cell cycle by lactate [104]. In liver cancer, CCNE2 lactylation induces the G1-S phase transition and promotes tumor progression. Sirt3-mediated delactylation of CCNE2 leads to the apoptosis of liver cancer cells [179]. Acetylation is cell cycle-dependent, occurring from the S phase through late G2 phase. During mitosis, the acetyltransferase PCAF [P300/CREB (cAMP response element binding protein)-binding protein-related factor] acetylates cyclin A at Lys (54, 68, 95,112), resulting in its ubiquitination by the anaphase-promoting complex/cyclosome and subsequent degradation by the proteasome and G1-S transition [180]. However, acetylation can have varying functions depending on the protein. The acetylation at K134 of lamin B1 (LMNB1) slows down the G1-S transition of the cell cycle [181].

In tumors, changes in key upstream genes are crucial for tumorigenesis and progression. P53, a tumor suppressor, can be lactylated at K120 and K139 by AARS1, which impedes its liquid-liquid phase separation, DNA binding, and transcriptional activation, thus promoting tumorigenesis and progression [14]. On the other hand, the P53 protein can be acetylated and activate the transcription of the p21 gene [182]. The acetylation of P53 also promotes ferroptosis and cell arrest in tumor cells [105, 183]. Additionally, the lactylated c-myc protein shows higher stability, upregulate the expression of cyclin D1 [184]. The acetylation of c-myc at K148 and K157 mediated by P300 and K323 mediated by GCN5 induces the transformation of tumor cells, cell adhesion and survival ability [106]. The lactylation of YAP mediated by AARS1 promotes the formation of the YAP-TEAD complex to promote the proliferation of gastric cancer (GC) cells [185]. Same as lactylation, when YAP is acetylated, its complex with TEAD more potently activates transcription of cell cycle related genes like CCNE1 and AURKA [107]. This enables cells to pass G1/S and G2/M checkpoints more readily, promoting cell proliferation.

### DNA repair

In tumor cells, both lactylation and acetylation contribute to therapy resistance, with DNA repair being a key process influenced by both modifications. Lactate influences DNA repair in multiple ways. Lactylated XRCC1 shows a stronger affinity for importin α, promoting its nuclear translocation and enhancing DNA repair [186]. XRCC1 is important participating molecules in base excision repair (BER). While in another report, deacetylation of XRCC1 mediated by SIRT1 stabilizes XRCC1 and promotes chemoresistance in lung cancer [187]. Recently, a series reports has provided evidence of lactylation influencing DNA homologous recombination repair (HR). The MRN complex is one of the earliest protein complexes recruited to DNA double-strand break sites [108]. It consists of NBS1, MRE11, and RAD50. MRE11 is a nuclease-active protein that is prominent in recognizing and processing DNA damage sites. Rad50 has ATPase activity and NBS1 interacts with multiple proteins, is crucial for signal transduction. The lactylation of K673 of MRE11 mediated by CBP promotes its binding to DNA, facilitates DNA end resection and HR [109]. Inhibiting this DNA repair effect makes cancer cells sensitive to cisplatin and PARPi. The lactylation of NBS1 at K388 is crucial for the formation of the MRE11-RAD50-NBS1 (MRN) complex and the accumulation of HR repair proteins at DNA double-strand break sites, mediating the radio-chemotherapy resistance of gastric adenocarcinoma. This effect was found to be mediated by TIP60 and HDAC3 [188]. TIP60 was discovered to play an important role very early in HR induced by the MRN complex, but it is achieved by influencing histone acetylation. When DNA is damaged, the acetyltransferase Tip60/Kat5 can recognize the exposed nucleosome with the histone mark of H3K9me3, thereby being recruited to the damaged chromatin, and is shielded from dephosphorylation to maintain the activation state. The activated Tip60 can activate ATM through acetylation (K3016 in the fatc motif) and affect the phosphorylation of MRN [110]. The acetylation of MRE11 and NBS1 has not been reported yet. In recent reports, RAD51 lactylation can enhance the homologous recombination (HR) response and increase cisplatin resistance in ovarian cancer [64]. This process is regulated by GCN5. However, in reports on RAD51 acetylation, HDAC1-mediated deacetylation of RAD51 leads to the stabilization of RAD51 and the enhancement of homologous recombination (HR) [189]. In addition, acetylation has a two-sided effect on DNA repair. Classic H2AX acetylation mediates DNA repair protein recruitment, while acetylation of K134 in lamin B1 inhibits non-homologous end joining by blocking 53BP1 recruitment to damaged DNA, thus promoting lamina diseases and cancer [110, 181].

### Metabolic regulation

Lactylation and acetylation may be metabolically linked. From the PTM perspective, acetylation may inhibit excessive oxidative phosphorylation activation, preventing mitochondrial overload and excessive ROS production. For example, K156ac of ME2 promotes the metabolism of glutamine into lactate, enhancing the overall lactylation level in tumor cells [132]. In non - histone lactylation, c-Myc, snail1, and SOX9/10 regulate intracellular glycolysis. The lactylation of ABCF1 and NUSAP1 activates the HIF1A and c-Myc, creating a positive feedforward loop and facilitating HCC growth and lung metastasis [111, 112]. In intervertebral disc degeneration (IDD), glutamine supplementation reduces AMPKα lactylation, increases its phosphorylation, and promotes autophagy and OXPHOS [190], inhibiting the progress of IDD. Similarly, in intervertebral disc degeneration (IDD), acetylation regulates ACSL4 degradation through chaperone-mediated autophagy to alleviate disc degeneration, and its role is similar to that of lactylation [113].

During glycolysis, PKM2 catalyzes the conversion of phosphoenolpyruvate (PEP) to pyruvate while generating one molecule of ATP. Lactylation of K62 on PKM2 inhibits its tetramer-to-dimer transition, enhancing pyruvate kinase activity [191]. However, what is perplexing is that PKM2 K62la increases metabolic flux but inhibits glycolysis in macrophage. In contrast, acetylation of PKM2 promotes its lysosomal degradation, impedes glucose metabolism [114, 192].

Meanwhile, lactylation regulates glucose metabolic flux in the PPP and nucleotide metabolism. In cervical cancer, DCBLD1 K172 lactylation stabilizes DCBLD1, inhibits G6PD autophagic degradation, activates PPP and nucleotide metabolism, thus promoting cervical cancer progression [115]. However, also in HPV-positive cervical cancer, it was discovered that the lactylation of G6PD inhibits the formation of G6PD dimers, and suppresses the PPP pathway and cell proliferation [116]. As for the acetylated G6PD, we found that acetylation can activate (AcK89) and inhibit (AcK403, K235, K171) [117, 193–196]. It also regulates the formation of dimers and the distortion of the protein structure. The acetylation of lysine 488 of the pyruvate dehydrogenase complex X component (PDHX) occurs prevalently in hepatocellular carcinoma, disrupting PDC assembly and contributing to the conversion of most glucose into lactate [197].

Hypoxia-induced glycolytic raises PDHA1 and CPT1 lactylation [198]. PDHA1 and CPT1 lactylation inhibits OXPHOS by restricting acetyl CoA influx from pyruvate and FAO (fatty acid oxidation), respectively. DHA1 hyperacetylation promotes Fis1 K20la. Fis1 lactylation drives excessive mitochondrial fission, causing ATP depletion and mtROS overproduction [118]. While in liver, lactylation of PCK2 also appears to exacerbate the entry of mitochondrial fatty acids into the TCA cycle and OXPHOS, intensifying ROS generation and ferroptosis during IR [119]. The lactylation of Vps34 enhances the binding of Vps34 to Beclin1, Atg14L, and UVRAG, thereby increasing the lipid kinase activity of Vps34 [119, 122]. Vps34 lactylation promotes autophagic flux and endosomal-lysosomal transport, linking lactylation and fatty acid metabolism to autophagy. VPS34 K29 acetylation inhibits PIK3C3-BECN1 interaction, while K771 acetylation reduces the affinity of PIK3C3 for phosphatidylinositol (PI), suppressing autophagy initiation [122]. Different from lactylation, the acetylation of VPS34 inhibits autophagy and lipid droplet clearance in lipid metabolism [120]. Using the alkyne - functionalized bioorthogonal reporter YnLac, PPAR1 lactylation was verified. This modification may regulate its ADP - ribosylation activity and glycolipid metabolism [55]. As an important key enzyme for de novo fatty acid synthesis, the lactylation at the K673 site of fatty acid synthase FASN inhibits the activity of fatty acid synthase, which mediates the downregulation of MPC1 on liver lipid accumulation [199]. Lactate was considered to promote de novo lipid synthesis in earlier reports [121]. During the development of Drosophila larvae, the acetylation of FASN inhibits de novo synthesis of transitional fatty acids within the larvae [200]. Above all, there may be a negative feedback between lactylation and de novo lipid synthesis to maintain intracellular fat homeostasis, for example, enhancing the lactylation of MeCP2 K271 increases M2 macrophage infiltration, promotes plaque stability, and reduces the risk of atherosclerotic cardiovascular disease [201].

In addition to glycolipid metabolism, lactylation also has regulatory effects on other metabolisms. In nicotinamide metabolism, the lactylation of NMNAT1 enhances the NAD+ salvage pathway mediated by enhancing its nuclear localization and maintaining enzymatic activity [129]. This lactylation support for the NAD+ salvage pathway in the glucose deprivation environment promotes the proliferation and survival of pancreatic cancer under energy deprivation. In liver cancer, AK2 (adenine kinase 2) is mainly located in mitochondria. Under energy stress (e.g., hypoxia or nutrient deficiency), AK2 activity is essential for maintaining AMP and ADP levels. The lactylation of K28 of AK2 promotes its enzymatic activity and promotes the proliferation and metastasis of liver cancer [6]. Similarly, the lactylation of IGF2BP3 is crucial for increasing the mRNA of PCK2 and NRF2, reprogramming serine metabolism and strengthening the antioxidant defense system, promoting lenvatinib resistance in hepatocellular carcinoma (HCC) [123]. PHGDH, the rate-limiting enzyme for de novo serine synthesis, can support the expression and maturation of IL-1β by mediating the transcriptional activation of Toll-like receptor 4 through H3K9/27 acetylation and the activation of the NLRP3 inflammasome through NLRP3-K21/22/24/ASC-K21/22/24 acetylation [152]. P300/CBP directly acetylates Nrf2, which increases the promoter-specific DNA binding of Nrf2 [124]. Lactate treatment significantly enhances the interaction between NSUN2 and the lactyltransferase NAA10, leading to enhanced lactylation and enzymatic activation of NSUN2, which then targets GCLC mRNA to promote m5C formation and mRNA stability, thereby inducing glutathione (GSH) synthesis [65]. The lactylation of GCLM exerts a similar effect of promoting GSH synthesis, protecting KRAS-mutant tumor cells from ferroptosis [35]. Iron metabolism is also involved in redox balance, especially related to lipid peroxidation and ferroptsis. K677 lactylation in tau protein may promote the progression of Alzheimer’s disease (AD) by influencing ferritinophagy and ferritin droptosis via the MAPK signaling pathway [202]. Therefore, the roles of lactylation and acetylation in ferroptosis are worthy of discussion. In cancer cells, nicotinamide nucleotide transglycohydrolase (NNT) is acetylated at lysine 1042 (NNT K1042ac), increasing the binding affinity of NNT for NADP + to enhance NNT activity, thereby promoting the production of NADPH, thus maintaining sufficient iron-sulfur clusters and protecting tumor cells from ferroptosis [131]. While the acetylation at different sites of P53 or other proteins shows different roles in ferroptosis [183, 203].

Lactylation of histone deacetylase (HDAC) may enhances its enzymatic activity and regulates genes associated with ferroptosis. Vorinostat (SAHA) and Trichostatin A (TSA) can decrease the HDAC1 K412la and contribute the ferroptosis susceptibility in CRC and KRAS mutant cancer [35, 59]. In another similar report, tRF-31R9J (an effective component of Tectorigenin) was identified to recruit HDAC1 to reduce the levels of histone lactylation and acetylation modifications of ferroptosis-promoting genes ATF3, ATF4, and CHAC1, thereby inhibiting their gene expression. But acetylated HDAC1 will lose its deacetylase activity [204]. Notably, HDAC1 function is regulated by its own modification status, as HDAC1 mediates the downregulation of histone lactylation and acetylation levels, and acetylation of HDAC1 abrogates its deacetylase activity, while lactylated HDAC1 may enhance its function. This modification-dependent functional divergence further validates the differential regulatory roles of lactylation and acetylation.

### Interactions with other PTMs

Here, we mainly discuss the roles of lactylation and acetylation in the regulation of ubiquitination and methylation. Modifications at the same site may have a competitive relationship, and most acyl modifications occur at the lysine site. Ubiquitination is a post-translational modification process of proteins, usually occurring at the lysine site [125]. Ubiquitin is a small molecule protein composed of 76 amino acids. Ubiquitination is an enzymatic reaction involving the participation of multiple enzymes, including ubiquitin-activating enzyme (E1), ubiquitin-conjugating enzyme (E2), and ubiquitin ligase (E3), which is involved in protein degradation, regulation of cell signal transduction, intracellular membrane transport, and maintenance of organelle homeostasis. The proteasome degradation system mediated by ubiquitination may be competitively inhibited by lactylation. As we discussed above, the lactylation of NUSAP1 and c-myc inhibits their ubiquitination levels, thereby increasing the stability and function of the proteins. NEDD4 lactylation inhibits protein interactions between Caspase-11 and NEDD4, inhibiting the ubiquitination of Caspase-11 [205]. In human primary pancreatic ductal adenocarcinoma (PDA) samples, the lactylation of TFEB at lysine 91 (K91) protects TFEB from ubiquitination and proteasomal degradation mediated by the E3 ubiquitin ligase WWP2, resulting in increased lysosomal activity and autophagic flux [126]. The acetylation of TFEB at residues K91, K103, and K116 (denoted as K91ac, K103ac, and K116ac), which is mediated by ACAT1, similarly activates autophagy. However, the acetylation of TFEB at residues K116, K274, and K279, mediated by GCN5, suppresses autophagy [130]. The lactylation of Serpina3k and LCP1was found to enhance protein stability and secretion, protecting the injury from ischemia-reperfusion injury (IRI) and ischemic stroke [136, 206]. Lactylation of ALDH2 promotes the ubiquitin-proteasome degradation of PHB2, thereby inhibiting mitophagy and exacerbating mitochondrial dysfunction [141].

The lactylation of the methylation regulatory protein METTL16 enhances the effect of cuproptosis inducers by increasing the m6A modification on FDX1 mRNA [133]. Two lactylation modification sites were identified in the zinc-finger domain of METTL3, which is essential for METTL3 to capture target RNA and participates in the regulation of the immunosuppressive microenvironment and DNA repair within the tumor [60, 127]. The role of acetylation in the methylation reaction is complex, but interestingly, METTL3 acetylation can regulate the subcellular localization of METTL3 and global acetylation, hindering metastatic dissemination. And lactylation may have the same effect [128]. MECP2 is an important protein for methylation recognition, and its lactylation can also play an important role in the methylation process [201].

### Others

Lactylation and acetylation have other important regulatory roles in cells. Here, we conduct a brief discussion. Lactylation may affect certain protein functions by regulating the liquid-liquid phase separation state. The lactylation (H131, M156) of cGAS can hinder the liquid-liquid phase separation (LLPS) of cGAS and inhibit the binding of cGAS to DNA [15]. The same phenomenon also occurs on P53, as we discussed above. The lactylation of P53 by AARS1 hinders its liquid-liquid phase separation, DNA binding, and transcriptional activation [14]. Near chromatin- proximal DSBs, combined acetyl groups on histone H2A (H2AK5acK9ac) prompt BRD4 to accumulate excessively [207]. BRD4 then undergoes LLPS with KU80, blocking proper LIG4-XRCC4-XLF installation at DSB ends, thus inhibiting NHEJ repair and genomic stability. While lactylation-induced LLPS of SORBS3 enhances its interaction with flotillin 1 and selectively promotes the sorting of F-box protein 2 (FBXO2V) into small extracellular vesicles termed “lactylosomes” [167]. Acetylation likewise inhibits the formation of liquid - liquid phase separation (LLPS). The acetylation of IRF3/7 and DDX3X each diminishes their respective LLPS [208].

Lactylation and acetylation also affect the secretion of certain molecules. For example, in macrophages, the lactylation or acetylation of HMGB1 may promote the secretion of HMGB1 [100]. The lactylation of Serpina3k at lysine 351 was found to enhance protein stability and secretion [206]. Lactic acidemia promotes the release of macrophage-derived CIRP by inducing its lactylation [209]. In turn, CIRP mediates ZBP1-dependent pan - apoptosis of pulmonary vascular endothelial cells (PVECs) in sepsis-induced acute lung injury (ALI). Lactylation of histone H3K18 and Egr1 promotes the degradation of the endothelial glycocalyx in sepsis-induced acute lung injury [135].

Protein lactylation can affect the binding of proteins to other molecules or proteins. In α-myosin heavy chain α-MHC as actin, promoting the lactylation at position K1897 of α-MHC and the interaction between α-MHC and Titin, thereby alleviating heart failure [206]. The increase of K150la promotes the phosphorylation and nuclear translocation of Twist1 and further regulates the transcription of TGFB1, thereby inducing the fibrotic phenotype [142]. The acetylation of TWIST1 (K73, K76) and SPZ1 (K369, K374) mediates the SPZ1-TWIST1-BRD4 axis in EMT and its promoting role in tumor initiation and metastasis [137]. K612 lactylation of APP reduces and inhibits its binding to BACE1, thereby suppressing subsequent cleavage [210]. Conversely, it promotes the protein - protein interaction between APP and CD2-associated protein (CD2AP), thus accelerating the endosomal - lysosomal degradation pathway of APP and facilitating the progression of Alzheimer’s disease.

The lactylation of eEF1A2 was found to increase the protein function, promote an increase in translation elongation rate and enhanced protein synthesis, thereby promoting tumorigenesis [140]. In another independent report, acetyl-CoA was found to inhibit translation in Escherichia coli, and further investigations showed that acetylation did not change the translation elongation rate, but increased the proportion of dissociated 30 S and 50 S ribosomes [138].

There are other reports on other roles of lactylation. In astrocytes, LRP1 inhibits glucose uptake, glycolysis, and lactate production, resulting in a reduction in the lactylation of ARF1, which may reduce mitochondrial transfer to damaged neurons and exacerbates IRI in a mouse model of ischemic stroke [211]. LCP1 lactylation has a certain correlation with the hydrocephalus score, which may be a feedback protective effect [136]. Acetylation also exhibits a protective effect on the nervous system. The acetylation of MIF (macrophage migration inhibitory factor) K78 inhibits the interaction between MIF and AIF, which weakens the translocation of MIF to the nucleus in ischemic cortical neurons and protects neurons from ischemic injury [139]. HDAC6-catalyzed α-tubulin-K40la enhances microtubule dynamics and promotes neurite outgrowth and branching in cultured hippocampal neurons [58]. In another study on skin fibrosis, acetylation of α-tubulin at the same site is essential for fibroblast activation, including contraction, migration, and extracellular matrix (ECM) deposition [212]. Interestingly, HDAC6 can catalyze lactylation, yet the specific reasons remain unclear.

## Difficulties in lactylation research

**Fig. 5:**
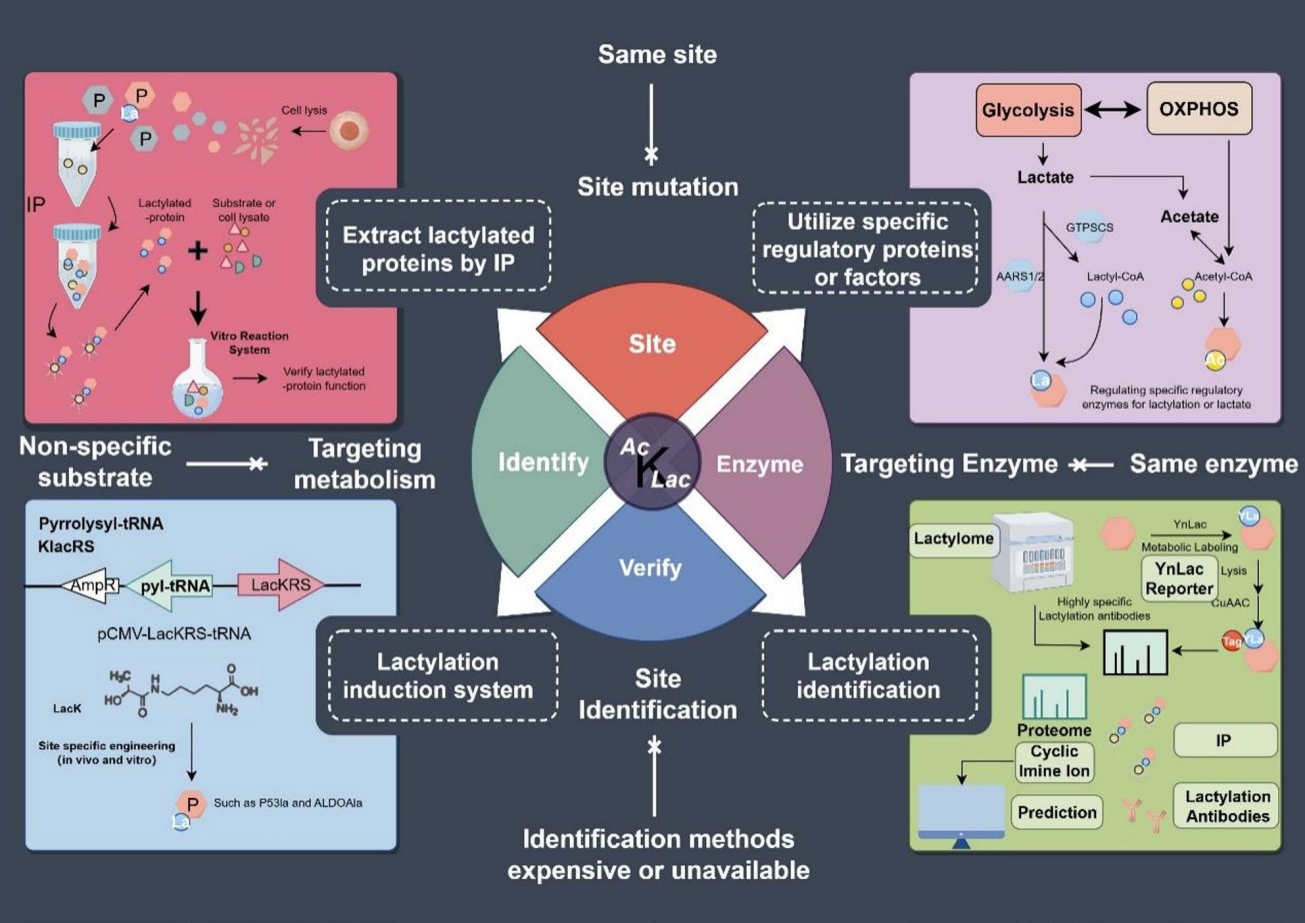
Challenges and Opportunities in the Research of Lactylation and Acetylation. Research on lactylation and acetylation faces certain difficulties as there is a lack of specificity in their regulatory mechanisms, action sites, and metabolic substrates, and the identification methods are costly. However, more and more techniques and solutions are being applied to lactylation and acetylation research. We can (1) regulate through specific proteins or substrates, (2) extract lactylated proteins via immunoprecipitation (IP) for vitro experiments, and (3) use the Pyrrolysyl - tRNA orthogonal system to directly generate modified proteins to verify the functions of relevant modified proteins. Regarding lactylation identification (4), we can verify it through methods like website prediction, proteomics (lactylome, cyclic imine ion), Ynlac, etc., along with the most basic and essential IP and verification with specific antibodies. P: protein. The figure was drawn using Figdraw

Although lactylation research has received extensive attention, studies on lactylation still face many difficulties. Lactylation modification belongs to acyl modification, and finding the protein functions or structural changes mediated by lactylation becomes a challenge (Fig. 5).

First, the research on lactylation generally initially focuses on the changes in the corresponding phenotype caused by lactate. However, the use of lactate in experiments does not directly imply changes in lactylation. As an important metabolic substrate, lactate can also be converted into pyruvate under the action of LDHB, enter gluconeogenesis or re-enter the tricarboxylic acid cycle through acetyl CoA [21]. Currently, to verify the effect of lactylation via lactate, researchers usually combine glycolysis and lactate - production genes or pharmacological inhibitors (e.g., oxalate, FX − 11, GNE − 140), along with mitochondrial inhibitors (e.g., rotenone) to prevent acetyl CoA formation or other metabolic impacts from lactate entering the TCA cycle, thereby demonstrating the lactate - mediated lactylation effect indirectly. Secondly, the effects of lactylation at various sites are verified by combining site - specific mutations with methods to enhance lactylation [15]. This verification method cannot rule out the situation of modification changes at the same site after lysine site mutation, such as acetylation, ubiquitination, and other acyl modifications occurring at the lysine site. Mutating lysine (K) to glutamine (Q) is a commonly used means for compulsory acetylation at the site. This method is theoretically the same as eliminating the negative ions of lysine in lactylation and acetylation modifications, but it still cannot highlight the effect of lactylation. Third, the effects of protein lactylation are verified by influencing the functions of non-specific acyltransferases or deacylases, but this method still cannot get rid of the influence of other acyl modifications in downstream phenotypes, that is, it has non-specific effects. The above are the traditional verification methods of protein modification. In extensive reports on lactylation, there are many reports that cannot directly verify the specific functions of the target protein modification, which may lead to the conclusions of these reports being potentially limited or misleading. Fourth, with the development of lactylation peptide purification, it is not difficult to verify lactylation proteins by lactylation mass spectrometry [7]. Combined with the evidence of pulldown of the target protein by IP, the conclusion of lactylation occurring on a single protein can be confirmed. However, limited by the conditions of each laboratory, the use of specific antibodies may be restricted, and only the changes in lactylation under specific treatment conditions can be judged through IP experiments. Indirect evidence is not without value, but requires supplementation with additional supportive evidence. For studies adopting such strategies, using lysine-to-glutamine mutants, complementary more evidence (e.g., orthogonal translation system (OTS) or acyl-CoA/acyltransferase rescue assays) should be integrated to confirm that observed phenotypes are specifically caused by lactylation, not acetylation or mere loss of positive charge.

There are many difficulties in the verification of lactylation, but currently, there are also some means for the study of lactylation that can obtain relatively reliable evidence. Due to the regulatory means of lactylation still not going beyond the scope of acyl modification, the use of special means to verify the effect of protein lactylation is necessary. In the report of lactylation of cGAS, by designing specific lactylation antibodies, purifying the lactylated cGAS protein through multiple IPs, and conducting enzyme activity experiments of the lactylated cGAS protein in vitro [15]. This kind of experimental evidence is considered reliable here. Nowadays, the acquisition of lactyl CoA is not difficult. Due to the complexity of the metabolic effects of lactate, the effect of lactyl CoA is more targeted [2]. Finally, an inducible orthogonal translation system with pyrrolysyl-tRNA synthetase is the best method to selectively increase the lactylation level in a dose-dependent manner [213]. In previous reports, the orthogonal translation system (OTS) with pyrrolysyl-tRNA synthetase was used instead of adding lactate or overexpressing LDHA to enhance the lactylation of p53 at specific sites. These means are considered here to be able to verify the effect of lactylation of specific proteins. Moreover, even without performing new mass spectrometry tests, protein lactylation can be identified by mining publicly available human melting maps. Wan et al. reported that the cyclic imine ion of lactyllysine formed during tandem mass spectrometry analysis can reliably determine protein lactylation [214]. A detection based on lactyl lysine-based cyclic imine ions and orthogonal translation system of pyrrolysyl-tRNA synthetase (PylRS) revealed extensive lactylation in the human proteome and lactylome, such as ALDOA and DHRS7, which may play a regulatory role in glycolysis [8, 213, 214]. Finally, if the conditions are limited, some lactylation prediction websites can be used, such as DeepKla and Auto-Kla [215, 216].

Monitoring acetyl-CoA, lactate, and lactyl-CoA in subcellular compartments enhances our understanding of physiological and pathological processes, with four primary approaches employed for this purpose. First, subcellular fractionation combined with targeted detection directly isolates specific organelles for metabolite analysis, offering simplicity and directness [217]. Second, isotope labeling and metabolic flux analysis utilize isotope-labeled metabolites (e.g., [U-13 C]lactate) to track metabolic flux in cells, and while this indirect method does not measure metabolite levels directly within organelles, it provides abundant and robust data by inferring organelle-specific states through the analysis of pathway-specific metabolites and their biological contexts—for instance, Bartman et al. mapped TCA cycle flux in various tumor models using intravenously administered [U-13 C]lactate coupled with imaging mass spectrometry (IMS), revealing that 13 C-lactate-labeled TCA intermediates were more abundant than those labeled by 13 C-glucose in the brain, a pattern distinct from other tissues [218–220]. Third, subcellular-targeted fluorescent probe imaging involves either incorporating fluorescent moieties into metabolites or designing probes that specifically bind target molecules [221–223]. Acetyl-CoA probes are widely developed, while lactate/lactyl-CoA probes (e.g., fluorescently labeled L-lactate analogs for investigating transport and metabolism) have also been reported, offering ease of operation with some probes enabling in vivo monitoring—Li et al., for example, developed a high-performance imaging technique (FiLa) for in situ, real-time, and quantitative tracking of lactate metabolism in live cells, subcellular compartments, and organisms, finding that nuclear lactate concentration is comparable to that in the cytoplasm, while mitochondrial lactate levels are significantly higher [224, 225]. Fourth, indirect distinction can be achieved via pathway-specific regulatory strategies such as omics approaches: single-cell metabolomics, for instance, captures metabolic changes in individual cells and infers intercellular metabolic crosstalk, providing the most comprehensive information but with weaker evidential power that requires validation through additional direct experiments [222, 223].

## Lactylation-targeted therapeutic strategies

Current intervention strategies for lactylation and acetylation mainly fall into three categories: targeting key metabolic proteins, directly regulating metabolite levels, and targeting modification enzymes. Extensive classic reviews have been published on targeted acetylation therapy; herein, we focus primarily on targeted lactylation therapy, especially its specific targeting approaches. Among these strategies, targeting key proteins in lactate metabolism centers on inhibiting lactate production and transport, with major targets including lactate dehydrogenase (LDHA/B) and monocarboxylate transporters (MCTs).

LDHA inhibitors have been widely investigated for cancer therapy. For example, Stiripentol, an antiepileptic drug approved by the FDA and EMA, has been identified as a potent LDHA inhibitor. Following preclinical validation of its lactylation-disrupting effects [188], stiripentol has entered oncology trials and is currently undergoing a single-arm phase II trial (ChiCTR 2400083649) to evaluate its efficacy in combination with immunochemotherapy for patients with refractory peritoneal carcinomatosis, while maintaining central nervous system safety without inducing toxic damage. In laboratory settings, compounds such as FX-11, galloflavin, N-hydroxyindole derivatives, and GNE-140 have all been demonstrated to exert robust inhibitory activity against LDHA, and FX-11 was classified as AT-101 derivatives [226–228]. AT-101 (gossypol), a targeted therapy for EGFR-mutant cancers, has been used in phase I/II randomized clinical trials for advanced non-small cell lung cancer with radiation therapy, head and neck cancer, and metastatic castration-resistant prostate cancer, yielding potential clinical benefits (NCT 01003769, NCT 00988169, NCT 00286780, NCT 00540722) [229]. Given that LDHB phosphorylation is involved in lactate generation, combined inhibition strategies targeting both LDHA and LDHB hold greater application potential, with representative agents including NCI 006 and the LDH proteolysis-targeting chimera (PROTAC) degrader MS6105 [230]. This PROTAC has successfully achieved dual targeting of LDHA/LDHB, inhibited the proliferation of pancreatic cancer cell lines, and exhibited favorable plasma bioavailability in mice following intraperitoneal injection. In contrast, the classic LDH inhibitor oxamate has not been translated into clinical application. Metformin, an inhibitor of respiratory complex I, has been verified to suppress lactate production but exhibits broad target specificity. Nevertheless, the combination of oxamate (an LDHA inhibitor) and metformin has been shown to induce colorectal cancer ablation in vitro model [231].

Directly targeting lactate by catalyzing its degradation to reduce tissue lactate concentrations represent a direct lactylation therapy strategy. Lactate oxidase (LOx) can catalyze lactate decomposition, release H₂O₂ and recruit immune cells, thereby overcoming immunosuppression and sensitizing tumors to immunotherapy. When LOx is encapsulated in nanomaterials (e.g., metal-organic frameworks, MOFs) and combined with CRISPR-mediated SIRPα genome-editing plasmids activated by pyruvate, it can polarize macrophages toward the M1 phenotype, forming a systemic therapeutic modality that exerts promising efficacy in the orthotopic treatment of breast cancer [232–234]. In a related study, Yao et al. developed novel CoMnFe layered double oxide (LDO) nanosheets with multi-enzyme activity, which convert lactate to pyruvate accompanied by reactive oxygen species (ROS) generation during radiotherapy, significantly enhancing the efficacy of radiotherapy for uveal melanoma [234, 235]. 

Targeting lactylation modification enzymes holds inherent advantages for precise therapy. AARS1/2 are classic regulatory enzymes that distinguish lactylation regulation from acetylation regulation, targeting AARS1/2 may thus help to elucidate the specific role of lactylation in various disease models. Alanine, the natural substrate of AARS1, can compete with lactate for binding to AARS1, thereby inhibiting AARS1-mediated lactylation and its downstream signaling pathways [14, 185]. However, in tumors, alanine also serves as a critical nutrient to promote tumor progression, and monotherapy with alanine has not demonstrated clear anti-tumor effects associated with lactylation inhibition [236]. Additionally, attention should be paid to the potential risk that AARS1 inhibition may disrupt protein translation and trigger unpredictable side effects [237]. Strategies targeting lactylation sites can also be achieved by designing small-molecule peptides to block specific lactylation sites or protein-protein interactions, providing a novel direction for combination therapy. For instance, the small-molecule peptide K673-pe can inhibit non-histone lactylation at the MRE11 K673 site in colon cancer, block DNA homologous recombination repair, and restore the sensitivity of tumor cells to chemotherapy and PARP inhibitors (PARPi), exhibiting a synergistic anti-tumor effect when combined with chemotherapy [109]. Similarly, D34-919 blocks the interaction between ALDH1A3 and PKM2 in glioblastoma multiforme (GBM) cells, inhibits XRCC1 downstream lactylation, and thereby restores the sensitivity of GBM organoids to temozolomide (TMZ) chemotherapy and radiotherapy [186].

## Summary and discussion

Both lactylation and acetylation are important regulatory mechanism in regulating cell fate. Cellular metabolites have complex effects on the internal activities of cells, and these effects are now associated with epigenetic modifications and post-translational modifications. The regulation and functions of lactylation and acetylation are highly similar yet different. The first thing to distinguish is the substrate changes caused by the corresponding metabolic fluxes. Lactylation and acetylation use lactyl CoA and acetyl CoA as substrates respectively, and lactylation can also be involved in lactylation under the action of AARS1/2 with the participation of lactate and ATP [14, 15]. Therefore, the difference in the metabolic fluxes of lactate and acetyl CoA is an important response for judging the overall lactylation and acetylation of cells. In terms of glucose metabolism, the corresponding indicators of glycolysis and OXPHOS can be used as indirect indicators of lactylation and acetylation [24]. Second, highlighting the role of lactylation or acetylation by targeting the differences in the regulatory methods of the two modifications. Through our summary, we can find that lactylation and acetylation share the same regulatory system within the scope of acyl modification regulation. The discovery of the regulatory role of AARS1/2 on lactylation brings hope for the future discovery of more specific regulatory proteins for lactylation or acetylation [14, 15]. Third, distinguishing the essential differences between lactylation and acetylation modifications. Some progress has been made in this direction. In histones, P53, and other related phenotypes such as immune regulation, lactylation and acetylation show obvious differential effects, and these results have achieved relatively reliable results [14, 183].

Summarizing the differences between lactylation and acetylation emphasizes lactylation’s functional specificity, using acetylation as a representative of other modifications. Past acetylation - targeted research has been extensive but has shown contradictory results, likely due to differences in protein effects or acetylation sites. Even without considering conclusions, much past data fails to clearly demonstrate acetylation’s function. Thus, summarizing the differences, similarities, and research challenges of lactylation and acetylation not only reveals lactylation research hurdles but also offers new perspectives on past evidence for acetylation and other modifications. Promoting epigenetic and PTM research relies on new technology dissemination. While classic methods exist, they have limitations. For instance, in vitro experiments using specific antibodies to purify lactylated proteins to mimic intracellular conditions work well for known enzyme reaction systems but are less suitable for unknown systems or non - enzyme proteins, and lactylation antibody use isn’t widespread. The inducible orthogonal translation system based on pyrrolysyl - tRNA synthetase can synthesize lactylated proteins without antibodies, yet it still faces technical hurdles [213].

Advances in mass spectrometry, genomic engineering, and chemical probes offer new opportunities for lactylation research. This also provides a fresh view on acyl modification studies. Previously, less precise methods were tolerated in acyl modification research, but with the discovery of more acylations like lactylation, succinylation, and crotonylation, more effective methods are urgently needed. Fortunately, such methods have been developed and applied. As mentioned above, the orthogonal translation system, recognized as the “gold standard assay” for PTM validation, resolves the co-localization of lactylation and acetylation at the same site via forced incorporation of specific modifications at designated positions; in vitro protein function validation based on immunoprecipitation (IP) enables precise verification of specific acylation-regulating proteins or lactylation-modified proteins. These two technologies address non-specificity in most studies and, despite technical barriers and operational difficulties, hold promotional value for acquiring accurate data. Additionally, efficient extraction of lactyl-CoA and acetyl-CoA, along with customization of specific antibodies, simplifies their validation experiments. We anticipate the design and application of more precise experimental methods. In the future, acylation research will deepen, and potentially misleading past findings will be corrected.

## Data Availability

Not applicable.
